# Analysis of Common Treatment Drugs and Allergen Sensitization in Hair Loss Patients

**DOI:** 10.1111/jocd.16798

**Published:** 2025-02-18

**Authors:** Chengcheng Feng, Ying Wang, Dingquan Yang, Hui Shen

**Affiliations:** ^1^ Department of Dermatology Zhangjiagang TCM Hospital Afliated to Nanjing University of Chinese Medicine Zhangjiagang People's Republic of China; ^2^ Department of Dermatology China‐Japan Friendship Hospital Beijing People's Republic of China

**Keywords:** allergenic reactions, hair disorders, patch testing, personalized treatment, sensitizing agents

## Abstract

**Background:**

Hair loss significantly impacts an individual's appearance, self‐confidence, quality of life, and social competitiveness. Over 250 million people in China suffer from hair loss, accounting for 18% of the population. To ensure the safe selection of topical hair products for hair loss patients, reduce the risks of allergies and irritation, and improve clinical efficacy and adherence, we developed a hair patch test based on clinical needs.

**Objective:**

In this study, we explored the allergic situation of hair diseases through the test of patch products, which provided a strong basis for clinical personalized treatment.

**Methods:**

We designed a hair patch test to screen for allergens that are highly associated with hair diseases. The experiment is based on screening of haptens and drugs commonly encountered by hair and scalp, covering hair dyes, scalp care products, and therapeutic drugs. The research subjects are patients diagnosed with hair related diseases (such as androgenetic alopecia, alopecia areata, etc.), ensuring that the samples are targeted. The allergen patch is fixed on the surface of the skin, with a contact time of 48 h, followed by recording and evaluating local skin reactions within 72 h. The experiment aims to identify allergens that may cause or exacerbate hair diseases, providing a basis for the etiology research and treatment of related diseases.

**Results:**

Common allergens in daily hair care products include dodecyl polyglucoside (10.2% positivity rate) and Balsam of Peru (8.67% positivity rate). Cobalt chloride (56.63% positivity rate) and nickel sulfate (32.65% positivity rate) were prevalent metal semiallergens. The positive rates of 5% minoxidil liniment were 22.96%, and the positive rate of 5% minoxidil tincture was 27.04%, indicating significant sensitization potential. Lowering minoxidil liniment concentration to 2% reduced positivity to 4.08%. The positive rate of Hasonide solution mixed with 5% minoxidil tincture was 16.33%, while Hasinide solution and 5% minoxidil liniment were not positive, suggesting that the addition of both Hasonide solution could reduce the sensitization of 5% minoxidil tincture and minoxidil liniment.

**Conclusion:**

The increasing prevalence of hair disorders necessitates personalized treatment approaches. This study demonstrated the utility of patch testing with hair care products to investigate allergenic reactions, offering robust evidence for tailored clinical interventions. Future research should refine patch testing methodologies to better simulate real‐world conditions and conduct larger‐scale studies to optimize therapeutic strategies.

Hair loss significantly influences the individual's image, self‐confidence, quality of life, and societal competitiveness, as evidenced by its impact. According to statistics, approximately 20% of the global population suffers from various hair disorders. In China, the population affected by hair loss exceeds 250 million people, accounting for a high proportion of 18% of the total population [1]. Among these, androgenetic alopecia, telogen effluvium, and alopecia areata are the most predominant types. Hair disorders share common attributes with chronic conditions, medical esthetics, and dermatological diseases, necessitating long‐term comprehensive management. Topical therapy represents the primary approach for hair loss disorders [2, 3], encompassing topical medications, medical devices, and hair care products. However, when using these hair care products, local irritation and allergic reactions may occur due to primary ingredients, excipients, and individual differences [4], thereby potentially impacting clinical efficacy, safety, and patient compliance. The patch test, as a classic diagnostic method for assessing patients' allergic reactions to topical products and medications, determines whether patients are allergic to the components they come into contact with. It is widely utilized in the diagnosis of conditions such as contact dermatitis [5]. To ensure the safe selection of topical hair care products for patients with hair loss, minimize the risks of allergies and irritation, and enhance clinical efficacy and compliance, we have developed a patch test for hair care products tailored to clinical needs. The patch test for hair care products includes components such as allergens commonly found in daily scalp care products, metals from scalp‐contacting items, commercially available or hospital‐compounded topical scalp medications, and pharmaceutical combinations designed to address common sensitizing agents. Therefore, the patch test for hair care products is of significant importance for clinical safety, rational, and precise medication use, as detailed in the following report.

## 1 Materials and Methods

### 1.1 General Information

A total of 196 patients diagnosed with hair loss at our department from October 2023 to December 2023 were included in the study. Among them, there were 110 male and 86 female patients, with an average age of 32.34 ± 7.48 years (range: 7–56 years). Specifically, there were 135 cases of androgenetic alopecia, 15 cases of telogen effluvium, 37 cases of alopecia areata, and 9 cases of other types of hair loss. From the medical records and hair microscopic imaging data of 196 subjects, we found that 109 subjects had head‐seborrheic dermatitis, 146 patients had folliculitis, and no patients had contact or irritant dermatitis.

### 1.2 Methods

In this study, patch testing was conducted using 20 substances, including 7 scalp care product haptens, 2 metal haptens, and 11 commonly used topical medications and combinations (see Table 1 for specific names). The patch test employed a standard screening series of hapten diagnostic kits [produced by Chemo Technique Diagnostics, Sweden, and distributed by Beijing Yuankang Medical Technology Co. Ltd.]. Medications were used in their original liquid form, and procedures strictly followed manufacturer instructions. Haptens and medications were applied to normal skin on both sides of the patient's spine, firmly pressed to ensure allergen‐skin contact. The patch chambers were removed after 48 h, and after 30 min to allow for resolution of non‐specific erythema caused by pressure, the irritation response was assessed. Sensitization results were evaluated again after 72 h, with observation periods extended in special circumstances.

**TABLE 1:** Substances tested in hair care patch testing.

Semi‐antigen for care products	Metal semi‐antigen	Common hair drugs and combinations
Aromatic compounds I	Nickel sulfate	Selenium sulfide lotion
Alkyl polyglucoside	Cobalt chloride	Ketoconazole lotion
Methyl isothiazolinone+ Methylchloroisothiazolinone		Pi fukang Skin lotion
Benzyl alcohol		Minoxidil liniment 2%
Balsam of Peru		Minoxidil liniment 5%
Propylene glycol		Minoxidil liniment 5% + Hasinide solution
Dodecyl polyglucoside		Minoxidil tincture 5%
		Minoxidil tincture 5% + Hasinide solution
		Hair‐growth tincture
		Hasinade solution
		Compound clobetasol propionate ointment

### 1.3 Patch Test Evaluation Criteria

The results were assessed according to the criteria of the International Contact Dermatitis Research Group (IC‐DRG) (Table 2). Positive reactions with intensity at or above (+) are diagnostically significant. Irritant reaction (IR):Discrete patchy erythema, no infiltration. Extreme positive reaction(+++):Coalescing vesicles, ullous or ulcerative reaction. Strong positive reaction(++):Erythema, papulesInfiltration, discrete vesicles. Weak positive reaction(+):Erythema, papules, infiltration. Doubtful reaction(?+):Faint macular, no infiltration homogenous erythema. Negative (−): No skin changes. Refer to Table 2 for interpretation criteria.

**TABLE 2:** Criteria for result interpretation.

Symbol	Graphic expression	Implication	Skin performance
−	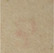	Negative	No skin changes
?+	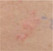	Doubtful reaction	Faint macular. No infiltration homogenous erythema
+	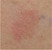	Weak positive reaction	Erythema·papules·infiltration
++	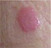	Strong positive reaction	Erythema. Papules infiltration·discrete vesicles
+++	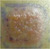	Extreme positive reaction	Coalescing vesicles bullous or ulcerative reaction
IR	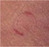	Irritant reaction	Discrete patchy erythema. no infiltration

### 1.4 Statistical Methods

The positive types and percentages of hair care patch test results in patients with hair disorders were analyzed using SPSS 22.0 statistical software, employing the chi‐square test. A significance level of *p* < 0.05 was considered statistically significant.

## 2 Results

### 2.1 Summary of Positive Results in Hair Care Patch Testing

Eleven types of topical hair care products were tested, including 3 shampoos, 2 ointments, 2 lotions, 1 solution, 1 cream, and 2 compound formulations. Seven cosmetic ingredients prone to sensitization were identified, categorized into 5 classes: preservatives, surfactants, fragrances, solvents, and metals, including 1 preservative, 2 solvents, 2 surfactants, 2 fragrances, and 2 sensitizing metals. The positivity rate and severity of allergic reactions are detailed in Table 3.

**TABLE 3:** Positive rate, severity of allergic reactions in hair care patch testing.

Sensibiligen	Positive (case)						
	?+	+	++	+++	IR	Number of positive samples	Positive rate(%)
Aromatic compounds I		11	1			12	6.12%
Alkyl polyglucoside		8				8	4.08%
Methyl isothiazolinone+ Methylchloroisothiazolinone		6				6	3.06%
Benzyl alcohol		3				3	1.53%
Balsam of Peru		16	1			17	8.67%
Propylene glycol		5				5	2.55%
Dodecyl polyglucoside		18	2			20	10.20%
Nickel sulfate		49	10	5		64	32.65%
Cobalt chloride		87	22	2		111	56.63%
Selenium sulfide lotion	1	48	8			57	29.08%
Ketoconazole lotion		88	9	1		98	50.00%
Pi fukang skin lotion		1				1	0.51%
Minoxidil liniment 2%		7	1			8	4.08%
Minoxidil liniment 5%	1	37	7			45	22.96%
Minoxidil liniment 5% + Hasinide solution		0				0	0.00%
Minoxidil tincture5%		49	4		1	53	27.04%
Minoxidil tincture 5% + Hasinide solution		30	2			32	16.33%
Hair‐growth tincture		4				4	2.04%
Compound clobetasol propionate ointment		12				12	6.12%

### 2.2 Results of Patch Testing for Common Hair Care Products in Hair Loss Patients

**FIGURE 1:**
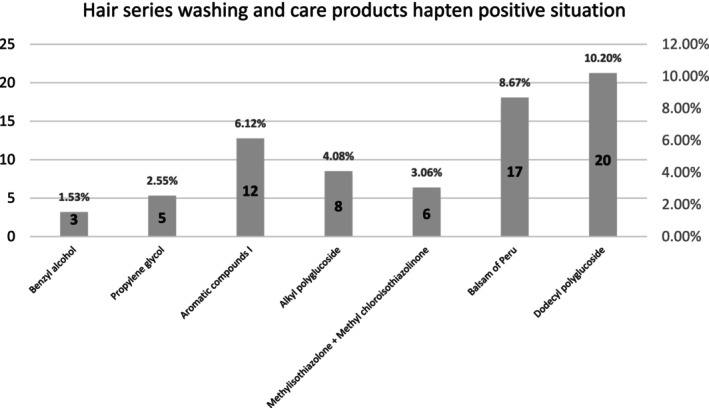
Patch test results of common hair care product haptens in patients with hair loss.

Our findings indicate that the positivity rate for standard haptens in hair care patch testing was 36.22% (71/196). The highest positivity rate among tested hair care product haptens was for dodecyl polyglucoside (10.20%), followed by Balsam of Peru (8.67%). Detailed test results are shown in Figure 1. Previous studies have shown that since 2012, the positivity rate for dodecyl polyglucoside has consistently remained above 1.5% (2% in 2012, 1.59% in 2013, 2.59% in 2014, and 2.7% in 2022) [6], the higher positive rate in this trial indicating a potential allergenic risk associated with dodecyl polyglucoside. The positivity rate for Balsam of Peru in Kuala Lumpur was reported as 7.1% [7], indicated that there is not much difference in the positive rate of Balsam of Peru between this study and Southeast Asia.

### 2.3 Patch Test Results of Metal Haptens in Patients With Hair Loss

**FIGURE 2:**
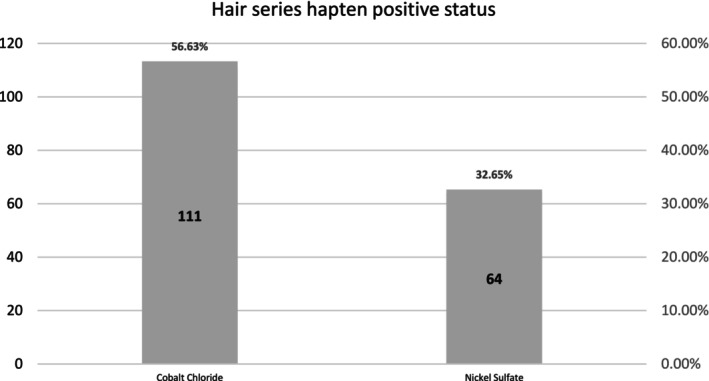
Patch test results of metal haptens in patients with hair loss.

In this study, the positivity rates for metal haptens cobalt chloride and nickel sulfate reached 56.63% and 32.65% respectively. The sensitization rate for cobalt chloride ranges was consistent with 51.46% of 1780 patients in the dermatology department of Beijing Air Force Characteristic Medical Center [8], higher 31.8% in Greece [9] and 33.3% in Poland [10], while the sensitization rate for nickel sulfate aligns with the 33% reported in America [10]. Specific positivity rate details are shown in Figure 2.

### 2.4 Allergic Testing Results of Common Topical Medications in Patients With Hair Loss

**FIGURE 3:**
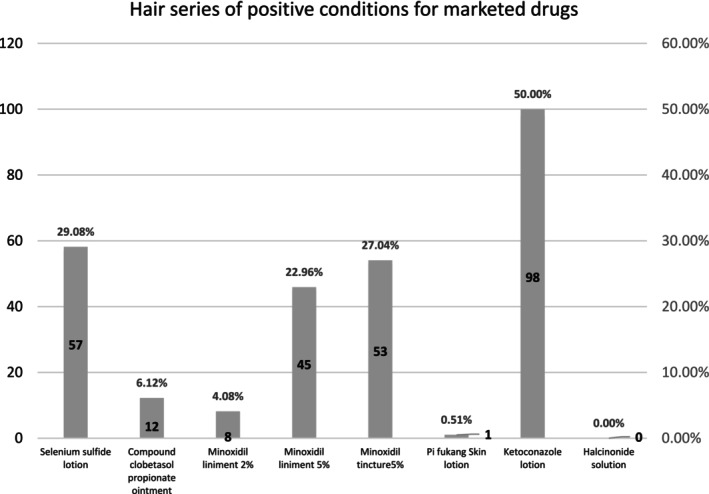
Allergic testing results of common topical medications in patients with hair loss.

Our findings indicate that the positivity rate for commonly used topical medications for hair loss was 74.49% (146/196). The highest positivity rate was observed for ketoconazole lotion (50.00%), followed by selenium sulfide lotion (29.08%). Specific details of the positivity rates are presented in Figure 3.

### 2.5 Allergic Testing Results of Topical Medication Interventions in Patients With Hair Loss

**FIGURE 4:**
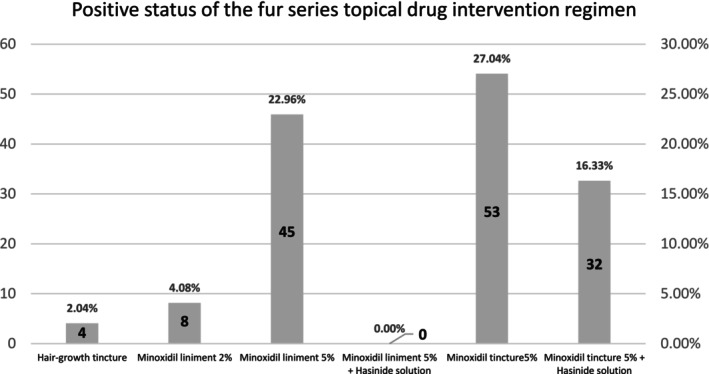
Results of allergy testing for topical medication intervention plans in patients with hair loss.

Our results show that among topical medication interventions, the highest positive rates were observed with minoxidil lotion and 5% minoxidil solution, at 27.04% and 22.96%, respectively. When combined with hydrocortisone solution, the positive rates were 16.33% and 0.0%, respectively. Specific positive rates are detailed in Figure 4.

## 3 Discussion

Patch testing is presently extensively utilized in the etiological investigation of conditions such as atopic dermatitis and contact dermatitis. Hair disorders are frequently associated with scalp seborrheic dermatitis, folliculitis, and medication‐induced contact dermatitis or irritant dermatitis. These conditions compromise the scalp barrier function, thereby influencing the application of topical treatments and the daily management of scalp and hair disorders. Based on the fundamental principles of patch testing and the commonly used scalp and hair care products and topical medications for hair disorders, we designed a hair product patch test. This innovation and extension of traditional patch testing protocols allows for preemptive elimination of safety risks associated with hair products, enabling precise selection of appropriate products for patients. It also provides a method to verify interventions for sensitizing products and ensures quality control for newly marketed topical medications. The hair product patch test significantly enhances patient compliance and clinical outcomes, thus holding substantial clinical significance and potential for widespread adoption.

Different countries have established their own standard allergens [10]. However, China currently lacks standardized allergens for contact hypersensitivity related to hair products. Consequently, we referred to the Chinese Baseline Series, Metal Series, Hairdressing Agents Series, and Skin Medication Adverse Reaction Series allergens, in conjunction with the European Society of Contact Dermatitis guidelines for diagnostic patch testing [11]. The primary sensitizing agents in routine hair care products include preservatives, surfactants, fragrances, and metals. Methyl isothiazolinone/Methylchloroisothiazolinone (MCI/MI) and benzyl alcohol serve as widely employed preservatives in cosmetics [12, 13, 14, 15]. Dodecyl polyglucoside is prevalent surfactants extensively utilized in hair care products such as shampoos and hair dyes [16, 17]. Hair care formulations commonly feature a substantial presence of fragrances, with aromatic compounds I and Balsam of Peru being predominant allergens [18]. Furthermore, common allergens reported in the literature to cause allergic contact dermatitis (ACD) on the scalp include cobalt metal in hair dyes [7]. Nickel‐containing alloy massage combs, applicators, microneedles, derma rollers, and hair accessories are also prevalent sensitizers upon contact. The organic solvent propylene glycol, commonly found in topical hair loss medications, is also an allergen [19]. Therefore, we selected nine common allergens from four categories of sensitizing agents and two metal allergens as patch test antigens for hair care products.

Topical minoxidil is the sole FDA‐approved medication for treating androgenetic alopecia [20, 21], yet local sensitization remains a prevalent adverse reaction. In our clinical experience, we have observed that combining 10 mL of hydrocortisone solution with 60 mL of minoxidil alleviates allergic symptoms in some patients sensitized to minoxidil. Selenium sulfide lotion and ketoconazole lotion, recognized for their efficacy in managing dandruff and seborrheic dermatitis, contribute to the rebalancing of scalp microbiota [22] and are widely utilized in patients with hair loss; however, some individuals may experience discomfort such as scalp dryness, itching, and increased scaling following use. Compound clobetasol propionate ointment, a compound formulation administered to severe alopecia areata patients, facilitates hair growth, and mitigates folliculitis adverse effects; nonetheless, some patients may encounter localized irritative responses during treatment. Additionally, Pi fukang Skin lotion, a frequently employed traditional Chinese topical wash in our department, exhibits anti‐inflammatory and anti‐pruritic properties. Our hospital's proprietary hair tonic formulation, known for its favorable outcomes, is also extensively employed; however, both formulations, being traditional Chinese compound preparations containing alcohol, may induce sensitization in certain patients. Thus, based on these clinical considerations, we selected nine topical hair loss medications and two combinations of topical treatments as allergens for this patch testing study on hair series medications.

Our study findings indicate that the most prevalent allergens in daily hair care products are dodecyl polyglucoside (positivity rate of 10.20%) and Balsam of Peru (positivity rate of 8.67%). Patients with sensitivity to these substances should avoid hair care products containing them. The metal semiallergens cobalt chloride and nickel sulfate showed positivity rates of 56.63% and 32.65%, respectively, indicating that individuals sensitive to these compounds should avoid all products containing them. Patch testing with topical medications revealed positivity rates of 27.04% for 5% minoxidil tincture and 22.96% for 5% minoxidil liniment, demonstrating a significant sensitization potential to minoxidil formulations, with similar concentrations showing comparable sensitization rates. In contrast, the positivity rate for 2% minoxidil liniment was only 4.08%, suggesting that lowering the drug concentration can reduce its sensitization potential. Furthermore, the combination of hydrocortisone solution with 5% minoxidil tincture showed a positivity rate of 16.33%, while no positivity was observed with the combination of hydrocortisone solution and 5% minoxidil solution, indicating that combining hydrocortisone with minoxidil can effectively mitigate allergic reactions.

## 4 Limitations

Nevertheless, limitations exist in our research; for instance, direct application of selenium sulfide lotion and ketoconazole lotion resulted in positivity rates of 29.08% and 50.00%, respectively, when used in the clinic, both lotions are often used in wet hair or mixed in proportion with the shampoo, which can reduce the true concentration of both lotions, potentially introducing errors in experimental outcomes. Future research should explore the impact of varying concentrations, formulations, and combinations of medications in patch testing to better simulate real‐world allergic reactions to hair care products, thus enhancing clinical diagnostic insights. Additionally, larger‐scale randomized studies employing standardized objective measurements are needed to compare the efficacy and safety profiles of different doses of hair care products, aiming to define optimal therapeutic strategies.

## 5 Conclusion

In conclusion, with the accelerating pace of modern life, the number of patients with hair disorders is increasing annually. However, addressing individual variability remains a pressing issue in achieving personalized treatment. This study utilized patch testing with hair care products to investigate allergenic reactions among patients with hair disorders, providing robust evidence for personalized clinical treatments. With advancements in hair disorder research and refinement of detection technologies, the application of patch testing in clinical diagnosis and treatment of hair disorders is expected to expand significantly.

## Author Contributions

Dingquan Yang and Hui Shen designed the experiments and revised the manuscript. Chengcheng Feng and Ying Wang carried outdata analysis, and writing and revising the manuscript. All authors revised and approved the final manuscript. Chengcheng Feng and Ying Wang contributed equally to this work.

## Conflicts of Interest

The authors declare no conflicts of interest.

## Data Availability

The data that support the findings of this study are available from the corresponding author upon reasonable request.
